# Belief in conspiracy theories: The predictive role of schizotypy, Machiavellianism, and primary psychopathy

**DOI:** 10.1371/journal.pone.0225964

**Published:** 2019-12-03

**Authors:** Evita March, Jordan Springer

**Affiliations:** 1 School of Health and Life Sciences, Federation University Australia, Berwick, Australia; 2 School of Health and Life Sciences, Federation University Australia, Ballarat, Australia; Western Sydney University, AUSTRALIA

## Abstract

A conspiracy theory refers to an alternative explanation of an event involving a conspirator plot organised by powerful people or organisations. Belief in conspiracy theories is related to negative societal outcomes such as poor medical decisions and a decrease in prosocial behaviour. Given these negative outcomes, researchers have explored predictors of belief in conspiracy theories in an attempt to understand and possibly manage these beliefs. In the current study, we explored the utility of personality in predicting belief in conspiracy theories. The aim of the current study was to explore the utility of the odd beliefs/magical thinking subtype of schizotypy, Machiavellianism, grandiose narcissism, vulnerable narcissism, primary psychopathy, and secondary psychopathy in predicting belief in conspiracy theories. Participants (*N* = 230; 44.7% male, 55.3% female) completed an anonymous, confidential online questionnaire which comprised demographics and measures of personality traits and belief in conspiracy theories. The total regression model indicated odd beliefs/magical thinking, trait Machiavellianism, and primary psychopathy were significant, positive predictors of belief in conspiracy theories. No other predictors reached significance. Results of the current study highlight individuals who might be more susceptible to believing conspiracy theories. Specifically, these results indicate that the individual more likely to believe in conspiracy theories may have unusual patterns of thinking and cognitions, be strategic and manipulative, and display interpersonal and affective deficits.

## Introduction

Conspiracy theories are a subset of false beliefs and generally implicate a malevolent force (e.g., a government body or secret society) involved in orchestrating major events or providing misinformation regarding the details of events to an unwitting public, in part of a plot towards achieving a sinister goal [1]. Although some conspiracy theories may be considered harmless, research has shown that belief in conspiracy theories is associated with negative social, health, and civic outcomes [2], including more acceptance of violent behaviour [3] and a decrease in prosocial behaviour [4].

Researchers theorise that belief in conspiracy theories addresses a need for certainty [5]. Traditional theories of opinion formation also explain these beliefs; specifically, individual predispositions, such as political and conspirator predispositions, have an effect on an individual’s reception of information cues [6]. Various individual differences have been found to predict belief in conspiracy theories, such as education [7], stress [8], boredom proneness and paranoia [9], and personality traits [10]. In the current study, we explore for the first time the utility of trait psychopathy to predict belief in conspiracy theories. Further, we explore the utility of dimensions of narcissism and psychopathy in predicting these beliefs.

Trait schizotypy, characterised by perceptual, cognitive, and affective abnormalities [11], has repeatedly been found to be a strong, positive predictor of beliefs in conspiracy theories [10, 12, 13]. Further, researchers [12] found the primary explanatory component for the relationship between schizotypy and belief in conspiracy theories was odd beliefs/magical thinking, as measured by a subscale in the Schizotypal Personality Questionnaire (SPQ). The tendency for an individual to hold unusual beliefs (i.e., odd beliefs/magical thinking) may explain the adoption of belief in conspiracy theories [12].

Although comparatively limited, research has found the traits of narcissism and Machiavellianism to be positive predictors of belief in conspiracy theories [14, 15]. Characterised by an inflated and grandiose sense of self [16], researchers attribute the relationship between narcissism and belief in conspiracy theories to the tendency for these individuals to perceive themselves as being the centre of attention, and that the actions of others are intentional attacks against them [14]. Trait Machiavellianism is characterised by the tendency to exploit and manipulate other people [17], and researchers posit such individuals may be more likely to believe in government conspiracy theories as they themselves would be likely to conspire if they were in that position of power [15]. Although the relationship between Machiavellianism and belief in conspiracy theories was mediated by ‘willingness to conspire’ [15], this mediation may be questionable due to possible collinearity between the mediator and the outcome variable.

### The current study

In the current study, we sought to address two particular gaps in the literature exploring belief in conspiracy theories: (1) To explore the utility of psychopathy predicting conspiracy theory belief, and (2) to adopt advice from researchers who caution treating narcissism and psychopathy as unidimensional. First, trait narcissism and Machiavellianism, along with trait psychopathy comprise the Dark Triad of personality [16], three non-clinical noxious personality traits that share exploitative tendencies, a manipulative interpersonal style, and a grandiose sense of self-importance [18]. However, unlike trait narcissism and Machiavellianism, trait psychopathy has not yet been explored as a predictor of belief in conspiracy theories. There is rationale to expect predictive utility, as characteristics of trait psychopathy such as the tendency to be exploitative, manipulative, have a grandiose sense of self-importance, and social dominance orientation, have all previously been associated with belief in conspiracy theories [14, 15, 19].

In addition to exploring the utility of all Dark Triad traits in predicting belief in conspiracy theories, the current study will also address the potentially problematic treatment of these dark traits as unidimensional [20], when both narcissism and psychopathy are dimensional traits [21]. Narcissism is considered to comprise two primary forms: Grandiose narcissism, characterised by a grandiose sense of self and superiority [22, 23]; and vulnerable narcissism, characterised by a vulnerable sense of self, insecurity, and negative emotionality [22, 23, 24]. Characteristics of narcissism attributed to belief in conspiracy theories, such as an inflated view of oneself requiring external validation [25], may better align with vulnerable, compared to grandiose, narcissism.

Trait psychopathy is also considered to comprise two forms: Primary psychopathy, characterised by social dominance, increased confidence, selfishness, lack of caring, manipulation of others, and a callous nature [26]; and secondary psychopathy, characterised by traits such as hostility, emotional reactivity, aggression, impulsivity [27] and a volatile interpersonal style [26]. Like trait narcissism, different dimensions of psychopathy may predict belief in conspiracy theories. Compared to the interpersonal and emotional detachment of primary psychopathy [27], secondary psychopathy is associated with higher levels of emotion dysregulation, greater impulsiveness and sensation seeking, and poorer interpersonal functioning [28]. The utility of primary and/or secondary psychopathy to predict belief in conspiracy theories will illuminate if such beliefs are better related to interpersonal dysfunctions and/or emotional dysregulation.

### Aims and hypotheses

The aim of the current study was to assess the utility of odd beliefs/magical thinking subtype of schizotypy, Machiavellianism, grandiose narcissism, vulnerable narcissism, primary psychopathy, and secondary psychology to predict belief in conspiracy theories. It was hypothesised that odd beliefs/magical thinking would be a significant, positive predictor of belief in conspiracy theories, and that Machiavellianism, grandiose narcissism, vulnerable narcissism, primary psychopathy, and secondary psychopathy would explain additional, significant variance.

## Method

### Participants

An initial 278 potential participants accessed the questionnaire. Of the 278, 28 did not progress past demographics and 20 did not complete all measures. The final sample comprised 230 participants (44.7% male, 55.3% female) with an average age of 26.54 years (*SD* = 7.48). Participants predominantly resided in Australia (44%) and were current university undergraduate students (43%).

### Measures

Materials were an online questionnaire including demographic questions (e.g., gender, age, country of residence) and measures listed in Table 1.

**Table 1:** Overview of measures included in the study questionnaire.

Title	Subscales	# of items	Example item	Scale	Current Cronbach’s *α*
MACH-IV [29]	N/A	20	Anyone who completely trusts anyone else is asking for trouble	1 = Strongly Disagree; 5 = Strongly Agree	*α =* .88
Brief-Pathological Narcissism Inventory (B-PNI [30])	1. Grandiose narcissism 2. Vulnerable narcissism	1. 12 2. 16	1. I often fantasise about being recognised for my accomplishments 2. When people don’t notice me, I start to feel bad about myself	0 = Not at all like me; 5 = Very much like me	1. *α =* .84 2. *α =* .89
Levenson Self-Report Psychopathy Scale (LSRP [31])	1. Primary psychopathy 2. Secondary psychopathy	1. 16 2. 10	1. Looking out for myself is top priority 2. I don’t plan anything very far in advance	1 = Disagree Strongly; 4 = Agree Strongly	1. *α =* .89 2. *α =* .77
Schizotypal Personality Questionnaire (SPQ [32])	Only odd beliefs/magical thinking subscale was included	7	Have you had experiences with the supernatural?	Yes/No	*α =* .79
The Generic Conspiracist Beliefs Scale (GCB-scale [1])	N/A	15	The government is involved in the murder of innocent citizens and/or well-known public figures, and keeps this a secret	1 = Definitely not true; 5 = Definitely true	*α =* .95

### Procedure

This project received ethical clearance from the institution’s Human Research Ethics Committee. Participants were recruited via an online advertisement posted on various social media sites (e.g., Facebook, Reddit) which advertised the anonymous, confidential online questionnaire. Participants were advised the questionnaire would take approximately 20–30 minutes to complete and gave consent to participate by ticking ‘yes’. Participants were not compensated for their participation.

### Design and analysis

This was a cross-sectional study with a correlational design. The predictors were odd beliefs/magical thinking, Machiavellianism, grandiose narcissism, vulnerable narcissism, primary psychopathy, and secondary psychopathy, and the criterion was belief in conspiracy theories. Gender and age were included in the regression analysis as control variables. To test the hypothesis, a 2-Step Hierarchical Multiple Regression Analysis was run. In accordance with recommendations [33] that reliable, established predictors are entered in the model prior to less established and new predictors, demographic controls of gender and age and predictor of odd beliefs/magical thinking were entered in Step 1. At Step 2, the predictors of Machiavellianism, grandiose narcissism, vulnerable narcissism, primary psychopathy, and secondary psychopathy were entered.

## Results

Descriptive statistics and bivariate correlations can be seen in Table 2.

**Table 2:** Descriptive statistics and bivariate correlations for variables of Machiavellianism, grandiose narcissism, vulnerable narcissism, primary psychopathy, secondary psychopathy, odd beliefs/magical thinking, and conspiracy theory beliefs.

	*M* (*SD*)	Range	1.	2.	3.	4.	5.	6.
1. Machiavellianism	64.27 (12.90)	42–97						
2. Grandiose narcissism	59.38 (21.29)	12–132	.62**					
3. Vulnerable narcissism	72.39 (30.07)	16–176	.71**	.75**				
4. Primary psychopathy	33.59 (9.52)	16–49	.66**	.41**	.54**			
5. Secondary psychopathy	23.36 (5.75)	10–35	.56**	.44**	.60**	.69**		
6. Odd/Magical	2.35 (2.19)	0–7	.53**	.44**	.52**	.51**	.54**	
7. Conspiracy beliefs	45.97 (14.86)	15–75	.67**	.49**	.60**	.61**	.53**	.61**

Odd/Magical = Odd beliefs/Magical thinking

*p < .01

**p < .001

Range = Min–Max

All predictors were positively, significantly correlated with the outcome variable and were therefore included in the Hierarchical Multiple Regression Analysis. At Step 1, odd beliefs/magical thinking, gender, and age explained 36.7% (R^2^ adjusted) of the variance in conspiracy theory beliefs, *F*(3,218) = 43.73, *p* = .001. At Step 2, Machiavellianism, grandiose narcissism, vulnerable narcissism, primary psychopathy, and secondary psychopathy explained an additional, significant 19.4% of variance, *F*change, (5,213) = 19.19, *p* = .001. The entire regression model explained 55.3% (R^2^ adjusted) of variance in conspiracy theory beliefs, *F*(8,213) = 35.23, *p* = .001. See Table 3 for coefficients.

**Table 3:** Coefficients for predictors of Machiavellianism, grandiose narcissism, vulnerable narcissism, primary psychopathy, secondary psychopathy, and odd beliefs/magical thinking .

	*B*	*SE*	*β*	*t*
*Step 1*				
Gender	-1.15	1.61	-.04	-0.71
Age	-0.18	0.11	-.09	-1.70
Odd/Magical	4.20	0.37	.61	11.41**
*Step 2*				
Gender	2.61	1.47	.09	1.78
Age	-0.06	0.09	-.03	-0.60
Odd/Magical	1.83	0.40	.27	4.53**
Machiavellianism	0.35	0.09	.30	3.91**
Grandiose narcissism	-0.02	0.05	-.01	-0.04
Vulnerable narcissism	0.06	0.04	.12	1.40
Primary psychopathy	0.34	0.11	.22	3.03**
Secondary psychopathy	0.05	0.18	.02	0.30

Odd/Magical = Odd beliefs/Magical thinking

*p < .01

**p < .001

Gender coded as 1 = Male, 2 = Female

As can be seen in Table 3, odd beliefs/magical thinking was a significant, positive predictor at Step 1 and remained a significant, positive predictor in Step 2. In addition, at Step 2 trait Machiavellianism and primary psychopathy were also significant, positive predictors of beliefs in conspiracy theories.

## Discussion

The aim of the current study was to assess the utility of odd beliefs/magical thinking, Machiavellianism, grandiose narcissism, vulnerable narcissism, primary psychopathy, and secondary psychology to predict belief in conspiracy theories. It was hypothesised that the odd beliefs/magical thinking subtype of schizotypy would be a significant, positive predictor of belief in conspiracy theories, and that Machiavellianism, grandiose narcissism, vulnerable narcissism, primary psychopathy, and secondary psychopathy would explain additional, significant variance; results partially supported this hypothesis.

As hypothesised, the odd beliefs/magical thinking subtype of schizotypy was a significant positive predictor of belief in conspiracy theories, thus corroborating previous research [10, 12]. Traits associated with unusual beliefs may lead an individual to believe in conspiracy theories [12], and the likeliness for individuals with high odd beliefs/magical thinking to hold delusional beliefs could explain their adoption of conspiracy theories [10]. Further, individuals with higher odd beliefs/magical thinking may seek a sense of control that belief in conspiracy theories could offer [34].

As predicted, Machiavellianism was a significant, positive predictor of belief in conspiracy theories, corroborating previous research [15]. As research on the relationship between Machiavellianism and belief in conspiracy theories is limited, interpretation of these results is somewhat speculative. Individuals with high trait Machiavellianism are strategic, exploitative, considered ‘master manipulators’ [35], and tend to have a cynical view of human nature [36]. As such, it is possible this these individuals believe other people to be foolish and easily manipulated; whereas they themselves are not so easily manipulated and know the truth. Another possibility is that due to this cynical nature and their tendency to manipulate others, individuals with high trait Machiavellianism might be hypersensitive to the possibility of being manipulated by those in power [15].

Contrary to the hypothesis, grandiose narcissism and vulnerable narcissism were not significant, positive predictors of belief in conspiracy theories. As previous research has found a relationship between trait narcissism and belief in conspiracy theories [14], it is possible that when assessed as dimensions, trait narcissism is no longer a significant predictor. However, this previous research assessed trait narcissism with a simplified version of the Narcissism Personality Inventory (NPI [37]), and the NPI is considered to measure grandiose narcissism [38]. As such, perhaps a more plausible speculation is that, upon inclusion of predictors that explain a significantly high percentage of variance in conspiracy theory beliefs (odd beliefs/magical thinking, Machiavellianism, and primary psychopathy explained 55.3% of variance); trait narcissism is simply no longer significant.

Results provided partial support to the prediction that trait psychopathy would predict belief in conspiracy theories. Interestingly, results showed only primary psychopathy was a significant (positive) predictor of belief in conspiracy theories. As discussed in the introduction, primary psychopathy is characterised by traits such as social dominance, self-confidence, selfishness, manipulation of others, and a callous nature [26, 27]. This more composed, confident nature of primary psychopathy contrasts the impulsive, destructive, and volatile nature of secondary psychopathy.

As characteristics associated with primary psychopathy such as social dominance, exploitation, and manipulation have all been associated with belief in conspiracy theories [14, 15, 19], it is not surprising that primary psychopathy was a significant positive predictor. The lack of utility of secondary psychopathy to predict belief in conspiracy theories suggests that such beliefs are less associated with impulsivity and emotional reactivity, and may underpin a careful, structured, and detached interpersonal style where relations with others are based on dominance and manipulation. This speculation is supported by the significant role trait Machiavellianism plays in predicting belief in conspiracy theories.

### Limitations and future directions

A possible limitation of the current study is that despite the internal consistency of the GCB-scale [1] being high (Cronbach’s *α* = .95), use of the GCB-scale is relatively limited. The GCB-scale was developed to address other measures of conspiracy beliefs being too geographically specific; specifically, the GCB-scale was developed as a more ‘general’ measure of conspiracy theory beliefs that was not bound by culture [1]. Despite this advantage, the scale has not been cross-culturally validated; a limitation relevant to the current study as individual characteristics and cultural factors are considered to interact when exploring belief in conspiracy theories [13]. Future research exploring belief in conspiracy theories should seek to include a culturally validated measure of conspiracy beliefs, such as the Conspiracy Mentality Questionnaire (CMQ [13]). As the CMQ only includes 5-items, the best approach would be to include the CMQ in addition to the GCB-scale.

Future research could also include measures of both generic (i.e., GCB-scale) and specific (Belief in Conspiracy Theories Inventory; BCTI [5]) beliefs in conspiracy theories. Inclusion of both measures could explore the viability of these measures, and if some traits are better predictors of specific conspiracy theory beliefs. For example, odd beliefs/magical thinking may be a better predictor of alien conspiracy theories compared to government conspiracy theories.

The current study may also be limited by not including a measure of self-esteem, as previous research has shown trait narcissism to interact with self-esteem to predict belief in conspiracy theories [14]. It is therefore unclear if grandiose narcissism and vulnerable narcissism were not significant predictors due to other, stronger predictors present, or if the interactive relationship with self-esteem was not measured. We recommend future research exploring narcissism and belief in conspiracy theories include a measure of self-esteem (specifically, the Rosenberg Self-Esteem Scale [39]).

Another potential limitation concerns the ecological validity of these results given that 43% of our sample were current university undergraduate students. Still, a number of studies exploring predictors of belief in conspiracy theories have examined samples that comprise only university undergraduate students [2, 7, 40]. We followed recruitment procedure of other researchers [1] exploring predictors of belief in conspiracy theories who attempted to recruit from the wider community. Unfortunately, the study [1] did not provide sample statistics regarding current university enrolments so a comparison is unable to be made. Although 43% of our sample reported they were current university undergraduate students, the sample was not recruited on campus and may be better representative of the wider population than previous studies exploring belief in conspiracy theories.

Finally, the current study is cross-sectional and correlational, so causality of belief in conspiracy theories cannot be established. However, results from a cross-sectional study may inform hypotheses for a more complex investigation [41]. Although beyond the scope of the current study, we recommend researchers use these initial results to guide future exploration of personality and belief in conspiracy theories, including interactions, possible mediations, and causal relations that exist amongst these predictors. Future research might also seek to explore belief in conspiracy theories as a possible ego-defence strategy [42] by inducing ego threat and then exploring the relationship between narcissism and these beliefs.

### Conclusion

Results of the current study indicate that individuals who have odd beliefs/magical thinking, and who are strategic, manipulative, dominant, and callous are more likely to believe in conspiracy theories. Given the possibility for these beliefs to shape real world behaviour, and the possibility for this behaviour to have damaging social and civic consequences, it is imperative research continues to establish predictors of these beliefs so reliable methods to challenge and negate these erroneous beliefs can be established.
